# Efficacy and safety of boosted darunavir-based antiretroviral therapy in HIV-1-positive patients: results from a meta-analysis of clinical trials

**DOI:** 10.1038/s41598-018-23375-6

**Published:** 2018-03-27

**Authors:** A. Antinori, A. Lazzarin, A. Uglietti, M. Palma, D. Mancusi, R. Termini

**Affiliations:** 1 HIV/AIDS Department, National Institute for Infectious Diseases “Lazzaro Spallanzani” IRCCS, Roma, Italy; 2 Department of Infectious Diseases, San Raffaele Scientific Institute, Milan, Italy; 3 Janssen-Cilag SpA, Medical Affairs Department, Infectious Diseases, Cologno Monzese, (MI) Italy

## Abstract

Darunavir/ritonavir (DRV/r) is a second-generation protease inhibitor used in treatment-naïve and -experienced HIV-positive adult patients. To evaluate efficacy and safety in these patient settings, we performed a meta-analysis of randomized controlled trials. We considered eight studies involving 4240 antiretroviral treatment (ART)-naïve patients and 14 studies involving 2684 ART-experienced patients. Regarding efficacy in the ART-naive patients, the virological response rate was not significantly different between DRV/r and the comparator. For the ART-experienced failing patients, the virological response rate was significantly higher with DRV/r than with the comparator (RR 1.45, 95% CI: 1.01–2.08); conversely, no significant differences were found between the treatment-experienced and virologically controlled DRV/r and comparator groups. Regarding safety, the discontinuation rates due to adverse events (AEs) and DRV/r-related serious adverse events (SAEs) did not significantly differ from the rates in the comparator group (RR 0.84, 95% CI: 0.59–1.19 and RR 0.78, 95% CI: 0.57–1.05, respectively). Our meta-analysis indicated that DRV/r-based regimens were effective and tolerable for both types of patients, which was consistent with published data.

## Introduction

Darunavir (DRV; TMC114; Prezista®) is a second-generation non-peptidomimetic protease inhibitor (PI) that was approved in 2007 in Italy for use in combination with ritonavir booster (DRV/r). DRV is used in combination with other antiretroviral (ARV) drugs for the treatment of human immunodeficiency virus (HIV) type 1 infection at two dosage regimens [800 mg once daily (OD) and 600 mg twice daily (both co-administered with ritonavir)]^1,2^. These regimens allow treatment of the entire setting of HIV-positive patients, from treatment-naive to highly experienced subjects and even those harboring HIV resistance mutations^3^.

The efficacy and tolerability of DRV/r have been evaluated in registrative randomized controlled clinical trials (RCT) in treatment-naïve^4,5^ and treatment-experienced^6–9^ patients with HIV-1 infection, with documented long-term efficacy and tolerability^7,10–12^. These results have been confirmed by real world evidence from observational studies^13^.

A once-daily co-formulation of DRV 800 mg plus a new booster, cobicistat 150 mg (Rezolsta®), is currently available. This fixed-dose combination (FDC) allows replacement of ritonavir as a booster for the treatment of both naïve and treatment-experienced adults^14^. The safety and efficacy of a single tablet regimen (STR) of darunavir/cobicistat/tenofovir alafenamide/emtricitabine (D/C/F/TAF) is being evaluated in two large phase III trials in treatment-naive and virologically suppressed patients (NCT02431247 and NCT02269917, respectively). The results of studies using cobicistat as a booster for darunavir showed no difference in efficacy from the use of ritonavir as a booster; therefore, the results of the present meta-analysis can be considered of interest even in this changing environment.

Current Italian^15^ (with some restrictions), European^16^, British^17^ and DHHS^18^ HIV/AIDS guidelines recommend the use of darunavir boosted with ritonavir or cobicistat as the only boosted protease inhibitor (bPI) (alongside other options, including integrase inhibitors and rilpivirine) as one preferred third agent in addition to a nucleoside reverse transcriptase inhibitor backbone, including tenofovir fumarate or tenofovir alafenamide and emtricitabine^18^.

Hence, the primary purpose of the present meta-analysis was to evaluate the efficacy, safety and tolerability of DRV/r-based regimens for treatment-naive HIV-1-infected patients or ART-experienced patients using reported RCTs.

## Results

**Figure 1:**
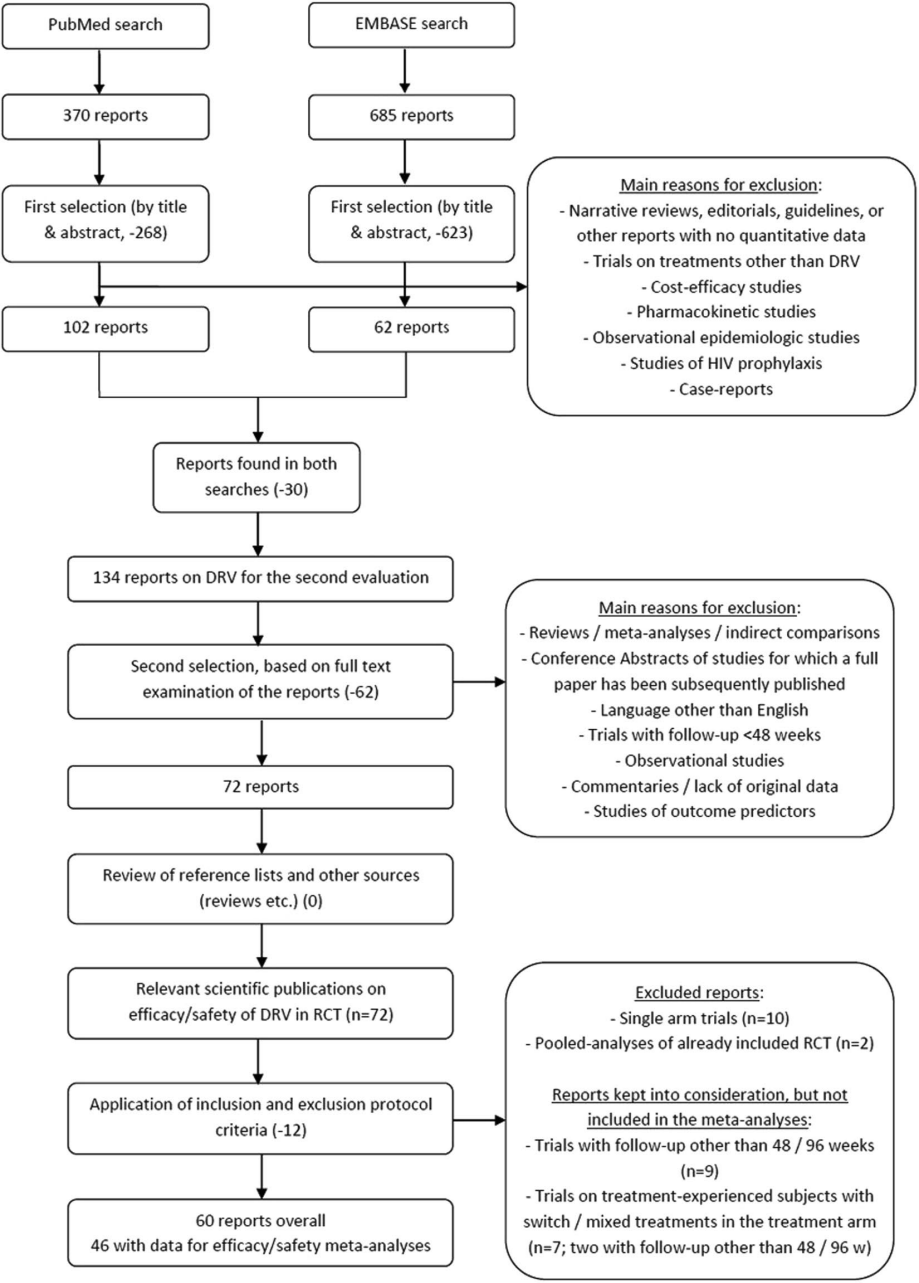
Flow-chart describing the literature search and study selection processes.

A search of electronic medical databases retrieved a total of 1055 articles. After title and abstract screening, we excluded 891 articles mainly because the authors did not report original data (i.e., narrative reviews, editorials, guidelines, or case reports) or the studies were designed as pharmaco-economic evaluations. After removal of duplicates using the Endnote X7 software, 134 articles on DRV were considered in-depth, and all full texts were downloaded and screened for final inclusion. After cross-checking for additional potentially missed references, 46 original articles with data on efficacy and safety were included in the present meta-analysis (Fig. 1). We considered three groups of studies based on the features of the enrolled patients: ART-naïve, ART-experienced failing and ART-experienced virologically controlled subjects. From a statistical perspective, we considered only studies with 48 and 96 weeks of follow-up (FU) to obtain sufficient subjects to conduct a meta-analysis. The main characteristics of the design and the baseline characteristics of the enrolled patients in the studies included in this analysis are summarized in Table 1 (ART-naïve adult patients) and Table 2 (ART-experienced adult patients). The results of the individual study quality assessments are reported and summarized in Supplementary Table 1. The study protocols were obtained where available to assess selective outcomes reports. The included studies achieved adequate sequence generation, but allocation concealment was not reported in all studies. All studies reported statistical analyses of the outcomes and addressed any incomplete data, such as loss to follow-up. All RCTs included were open-label; therefore, the two domains of performance bias and attrition bias were deemed to have a high risk of bias (Supplementary Table 1).

**Table 1:** Main characteristics of trials considering ART-naïve adult patients.

Trial	Reference(s)	Enrollment period	Geographic area	No. of enrolled subjects (treated:control)	Patient characteristics at baseline: -Median/mean age - Cutoff for plasma viral load (copies/ml) - Cutoff for CD4 cell count	Duration of follow-up (weeks)	DRV group regimen	Control group regimen
***ART*** *-* ***naïve patients (8 studies*** **;** ***4568 total patients enrolled)***								
ACTG 5257	Lennox JL^37^, Ofotokun I^43^	2009–2011	US & Puerto Rico	1809 (601:605:603)	-37 y (median) - pVL > 1000 -CD4 not limited	96	DRV/r (800 mg/d)	Two groups: 1) ATV/r 2) RAL
ARTEMIS	Ortiz R^4^	2005–2008	US, UK, Thailand, Argentina, France, Australia	689 (343:346)	-36 y (mean) in DRV/r and 35 (mean) in LPV/r -pVL ≥ 5000 -CD4 not limited	48	DRV/r (800 mg/d)	LPV/r
	Mills AM^5^					96		
	Lathouwers E^12^					96		
	Orkin C^10^					192		
ATADAR	Martinez E^44^	2011	Spain	178 (88:90)	-35 y (mean) treat vs 37 y (mean) control - pVL ≥ 1000 -CD4 not limited	96	DRV/r (800 mg/d)	ATV/r
FLAMINGO	Clotet B^20^	2011–2012	Europe, US and South America	484 (242:242)	-Adult -34 y (median age) - pVL > 1000 - CD4 not limited	48	DRV/r (800 mg/d)	DTG
	Molina JM^45^					96		
IMEA 040 DATA trial	Slama L^19^	2011–2013	France	120 (61:59)	-Adult -43 y (median) - pVL > 1000 - CD4 < 200	48	DRV/r (800 mg/d)	ATV/r
METABOLIK	Aberg JA^46^	NA	US	65 (34:31)	-36.5 y (median age) in the study group and 35.0 y in the control group -pVL > 1000 - CD4 not limited	48	DRV/r (800 mg/d)	ATV/r
NEAT001/ ANRS143	Raffi F^47^	2010–2011	Europe	805 (401:404)	-37 y (median age) in the RAL group and 39 y (median) in the TDF-FTC group -pVL > 1000 - CD4 < 500	96	DRV/r (800 mg/d) + TDF/FTC	RAL + DRV/R (800 mg/d)
OPTIPRIM-ANRS 147	Chéret A^48^	2010–2011	France	90 (45:45)	-35 y (median age) - pVL not limited - CD4 < 500	96	DRV/r (800 mg/d) + TDF/FTC	DRV/r (800 mg/d) + RAL/MVC + TDF/FTC

**Table 2:** Main characteristics of trials considering ART treatment-experienced adult patients.

Trial	Reference(s)	Enrollment period	Geographic area	No. of enrolled subjects (treated:control)	Reason for discontinuation of earlier treatments	Patient characteristics at baseline: - Median/mean age - Median/mean time since treatment started - Cutoff for plasma viral load (copies/ml) - Cutoff for CD4 cell count	Duration of follow-up (weeks)	DRV group regimen	Control group regimen
***Treatment****-****experienced failing subjects***, ***DRV 600*** ***mg BID (3 studies*;** ***1440 total patients enrolled)***									
ODIN	Cahn P^8^	NA	North, Central and South America, Europe, Australia and Asia	590 (294:296)	Treatment simplification	-40.2 y (mean age) in the study group and 40.7 y (mean) in the control group - pVL > 1000 - CD4 < 50	48	DRV/r (600 mg BID)	DRV/r (800 mg OD)
POWER (1–2)	Clotet B^22^	2005	Multicentric	255 (131:124)	Increase in drug resistance	-43.9 y (mean age) in the study group and 44.4 y (mean) in the control group - pVL > 1000 - at least one primary PI mutation	48	DRV/r (4 dosages; only 600 mg BID was included in the meta-analysis)	Control PI
TITAN	Madruga JV^9^	2005–2007	Multicentric	595 (298:297)	DRV experienced in the border range	- 40 y (mean age) - 9.1 y (mean duration of infection) - pVL > 1000 - CD4 not limited	48	DRV/r (600 mg BID) + OBR	LPV/r + OBR
	Banhegyi D^11^						96		
***Treatment***-***experienced***, ***virologically controlled subjects***, ***DRV 800*** ***mg*/*d (11 studies*;** ***1046 total patients enrolled)***									
2PM STUDY	Gianotti N^49^	2013–2014	Italy	43 (15:13:15)	NA	-Adult - 46 y (median age) - pVL < 50 - CD4 > 200	48	DRV/r (800 mg)	1) LPV/r 2) Triple
DRIVESHAFT	Huhn GD^50^	NA	NA	60 (30:30)	NA	- median age and previous ART duration are NA - pVL < 40 - CD4 not limited	48	DRV/r (800 mg OD)	DRV/r (600 mg BID)
DRV600	Moltó J^51^	2012–2013	Spain	100 (50:50)	NA	- 45.2 y (mean age) - 8.5 y (mean time since diagnosis) - pVL < 50 - CD4 not limited	48	DRV/r (800 mg)	DRV/r (600 mg)
LOPIDAR	Santos J R^52^	NA	Spain	75 (40:33)	Treatment simplification	- 43 y (median age) - 108 w (median HIV diagnosis) - pVL < 50 CD4nadir > 100	48	DRV/r (800 mg)	LPV/r
MIDAS	Hamzah L^53^	NA	NA	64 (32:32)	Side effects	-age NA - pVL < 50 - CD4 not limited	48	DRV/r (800 mg)	TDF/FTC/EFV
MONARCH	Guaraldi G^54^	NA	Italy	30 (15:15)	NA	- 45 y (median age) in the study group and 43 y (median) in the control group -pVL < 50 - CD4 > 200 - CD4nadir > 100	48 48	DRV/r (800 mg) + NRTIs (triple)	DRV/r (800 mg) monotherapy
MONET	Arribas JR^28^	2007–2008	Europe	256 (127:129)	NA	- 44 y (median age) - 7.4 y (mean) in art in the study group and 5.9 y in the control group - pVL < 50 - CD4 > 200	48	DRV/r (800 mg) + NRTIs	DRV/r (800 mg) monotherapy
	Clumeck N^55^						96		
PROBE	Maggiolo F^56^	2014	Italy	60 (30:30)	Avoid drawbacks and toxicities due to the nucleoside backbone	- 49 y (median) in the DRV group and 48 y in the control group - 93 m (median previous art) in the DRV group and 98 m in the control group - pVL < 50 - CD4 not limited - negative HBV	48	DRV/r (800 mg) + RPV	Triple
PROTEA	Antinori A^29^	NA	Europe and Israel	273 (137:136)	NA	- 42 y (mean age) -pVL < 50 for the previous 48 w - CD4 > 200	48	DRV/r (800 mg) + 2NRTIs (triple)	DRV/r (800 mg) monotherapy
	Girard PM^57^						96		
SPARE	Nishijima T^58^	2011	Japan	58 (28:30)	NA	- 44 y (median age) in the study group and 39 y in the control group - pVL < 50 CD4 not limited	48	DRV/r (800 mg) + RAL	LPV/r + TVD
***Treatment****-****experienced subjects***, ***mixed/other combinations (1 study; 225 total patients enrolled)***									
MONOI	Katlama C^30^	2007–2008	France	225 (112:113)	NA	- 46 y (median age) in the study group and 45 y in the control group -pVL < 50 -pVL < 400 for > 18 m	48	DRV/r (600 mg BID, switched to 800 mg OD if pVL < 50 at w48) + NRTIs (triple)	DRV/r (600 mg BID, switched to 800 mg OD if pVL < 50 at w48) monotherapy
	Valantin MA^21^						96		

NA: Not applicable

### Efficacy

Efficacy was defined as the virological response rate (viral load < 50 copies/ml) at 48 and 96 weeks for the ART-naïve adult patients and at 48 weeks for the ART-experienced patients.

**Figure 2:**
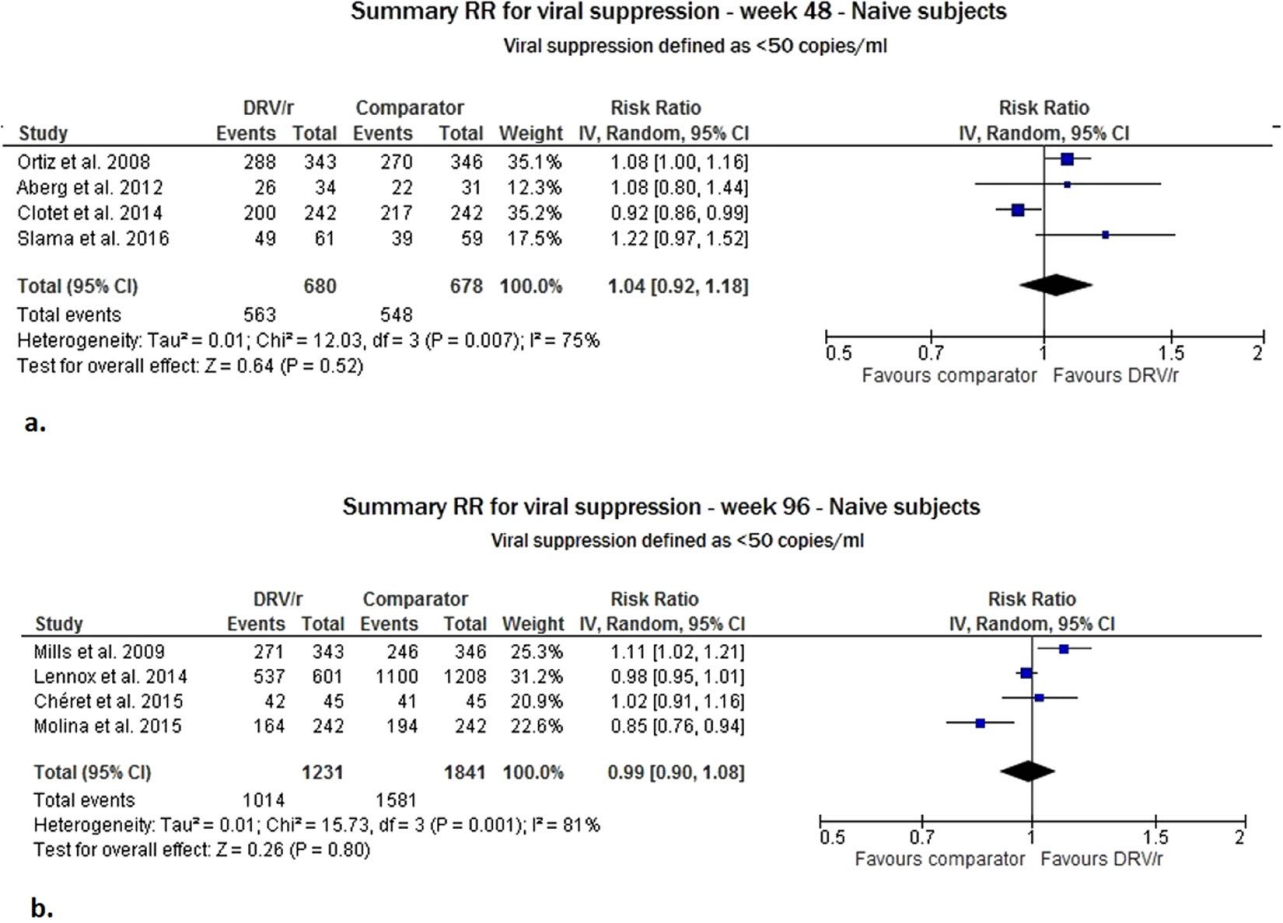
Meta-analysis of viral suppression for ART-naïve adult subjects at 48 (Panel a) and 96 (Panel b) weeks of follow-up.

For the ART-naïve patients, we included eight studies in this meta-analysis covering a total of 4430 adult patients evaluated (four with 48 weeks of FU and four with 96 weeks of FU). In the intention-to-treat (ITT) analysis, the virological response rate with DRV/r was not significantly different from the comparator at weeks 48 and 96, with risk ratio (RR) values equal to 1.04 (95% confidence interval (CI): 0.92–1.18) and 0.99 (95% CI: 0.90–1.08), respectively. A high degree of heterogeneity emerged between the RR estimates at week 48 (heterogeneity test I^2^ = 75%, p = 0.007) and week 96 (I^2^ = 81%, p = 0.001) (Fig. 2).

**Figure 3:**
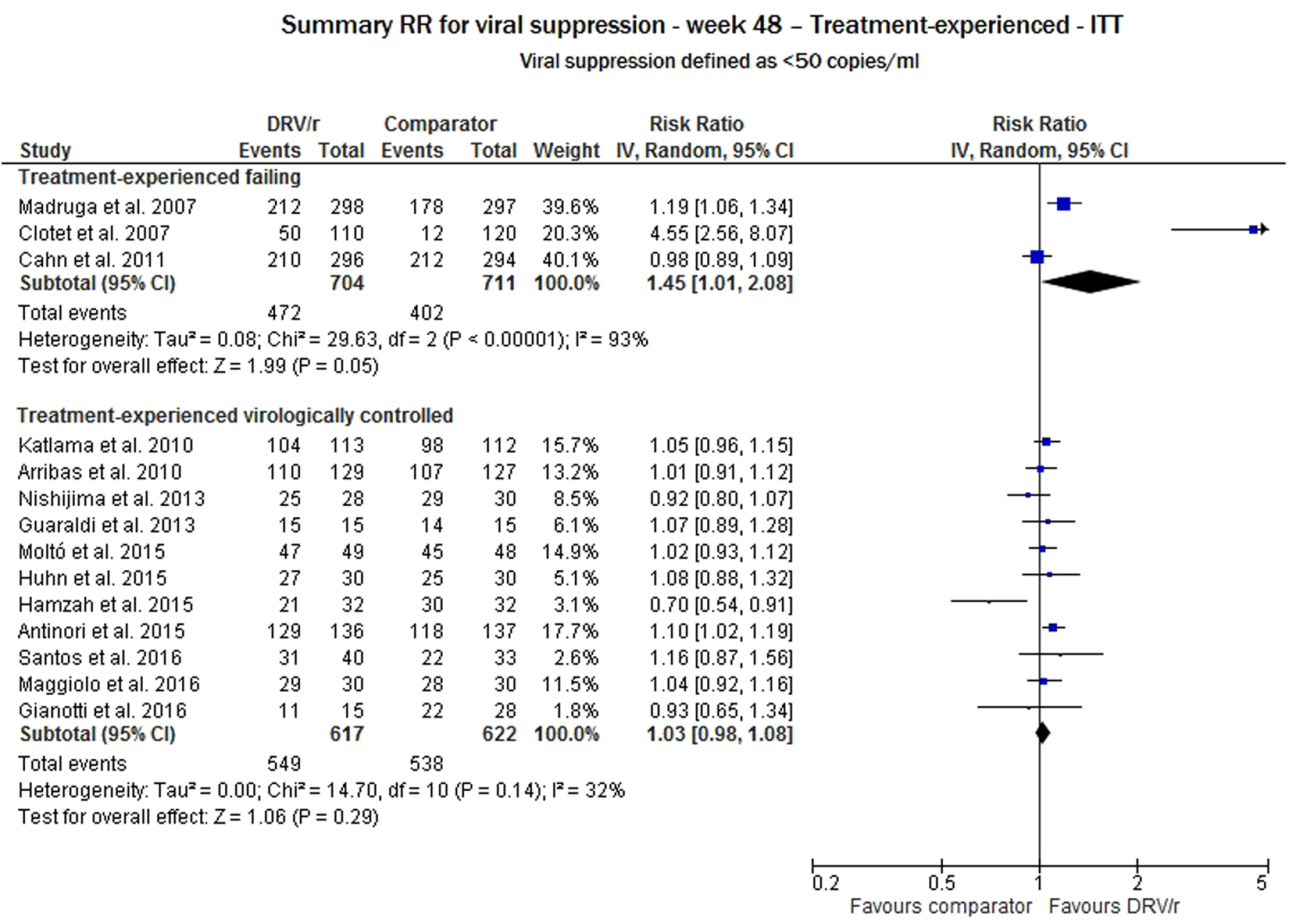
Meta-analysis of viral suppression for ART-experienced adult subjects at 48 weeks of follow-up.

For the ART-experienced patients, data were available from three studies for failing subjects (a total of 1440 adult patients evaluated) and from 11 studies for virologically controlled subjects (a total of 1553 adult patients evaluated). At week 48, the ITT analysis of the treatment-experienced failing subjects showed that the virological response rate was significantly higher for DRV/r than for the comparator group (RR 1.45, 95% CI: 1.01–2.08), but the heterogeneity test showed high variability among the studies (p < 0.0001). Conversely, for the treatment-experienced virologically controlled DRV/r group, no significant difference was found between the DRV/r and comparator groups (RR 1.03, 95% CI: 0.98–1.08), and the variability of the study estimate was low (I^2^ = 32%, p = 0.14) (Fig. 3).

In the sensitivity analyses conducted in naïve subjects at week 48, we calculated pooled RRs after excluding the studies one by one. No study had a notable influence on the overall estimate, because the pooled RRs varied between 1.01 (when excluding the IMEA^19^ study) and 1.09 (when excluding the FLAMINGO^20^ study). The same result was obtained for the treatment-experienced virologically controlled subjects. No evidence of publication bias was detected.

### Safety

**Figure 4:**
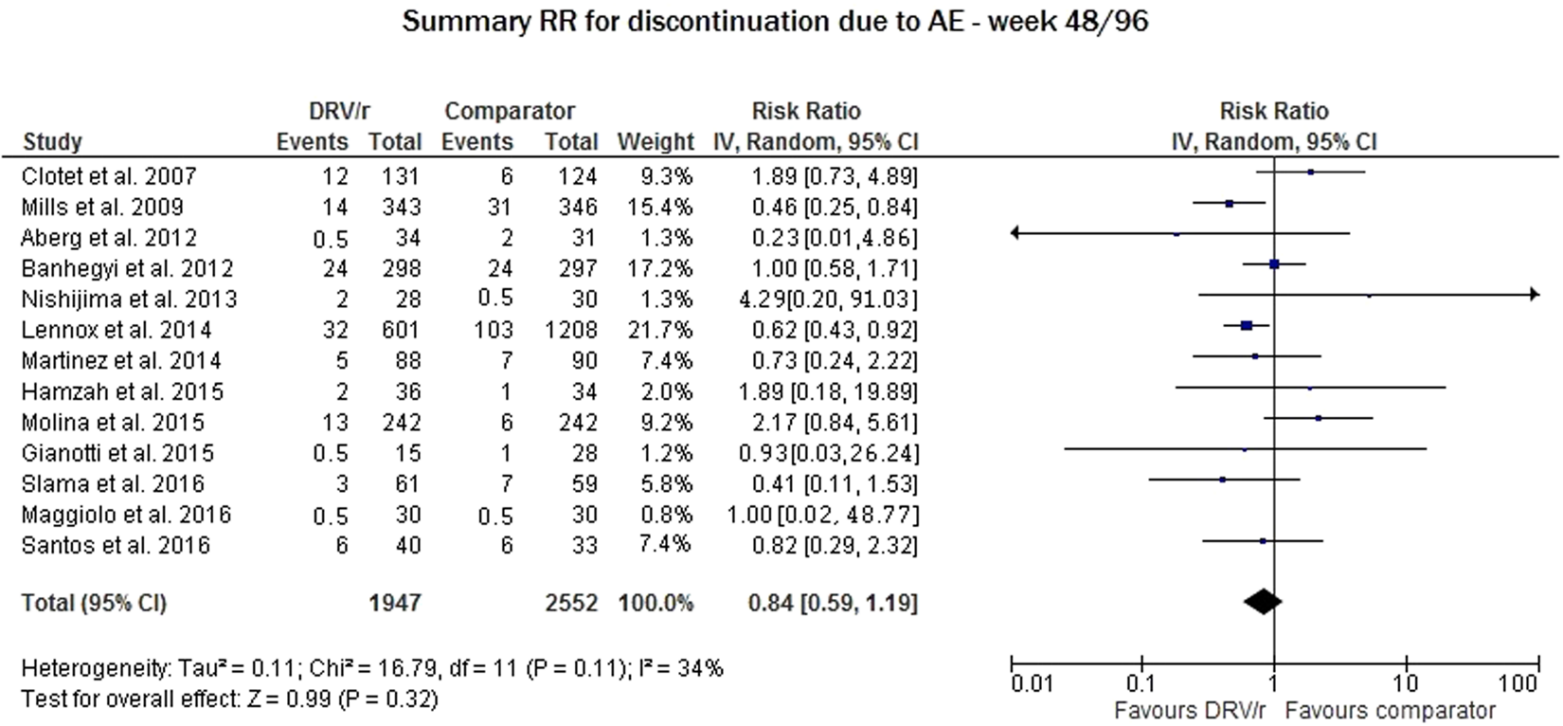
Meta-analysis of studies reporting data on treatment discontinuation due to adverse events and any serious adverse event related to the administered treatment.

We evaluated the discontinuation rate due to adverse events (AEs) related to DRV/r for 13 studies and pooled the results for weeks 48 and 96. The DRV/r safety profile was not significantly different from that of the comparator (RR 0.84, 95% CI: 0.59–1.19); this result was supported by the low variability between studies (I^2^ = 34%, p = 0.11), as shown in Fig. 4.

**Figure 5:**
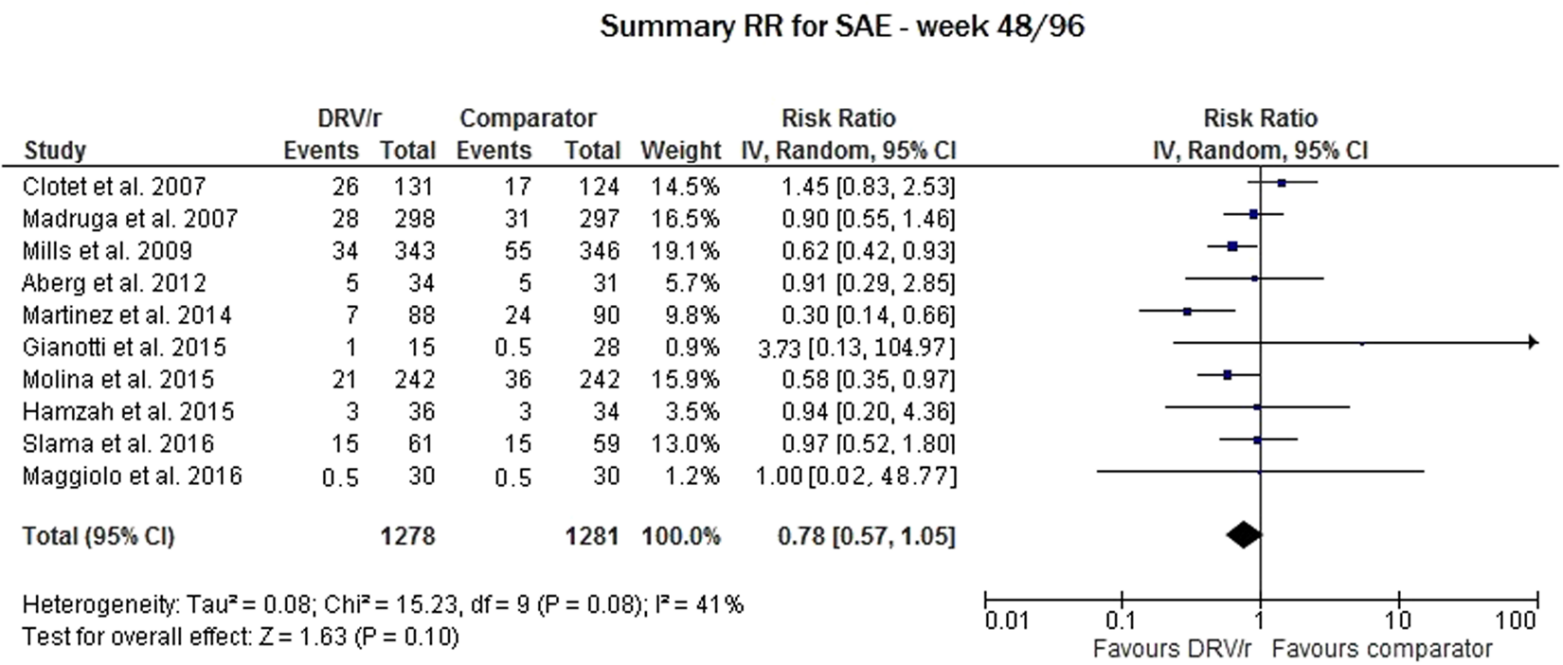
Meta-analysis of studies reporting data on any serious adverse events (SAEs).

Regarding the discontinuation rate due to serious adverse events (SAEs) related to DRV/r, we evaluated 10 studies and pooled the results for weeks 48 and 96. In this analysis, the difference between the DRV/r and the comparator was also not significant (RR 0.78, 95% CI: 0.57–1.05), and low-to-moderate variability was found between the study RRs (I^2^ = 41%, p = 0.08) (Fig. 5).

Notably, cardiovascular (CV) events were analyzed for all of the studies included in this meta-analysis. In the 19 trials (including a total of 4992 subjects), seven non-specified CV events were reported in the MONOI^21^ trial, one stroke was reported in the DRV/r arm and one myocardial infarction (MI) in the lopinavir/ritonavir (LPV/r) arm in the ARTEMIS^10^ trial, one MI was reported in the DRV/r arm and one cardiomyopathy in the dolutegravir (DTG) arm in the FLAMINGO^20^ trial, and one case of pericarditis was reported in the atazanavir (ATV) arm in the IMEA^19^ trial. When publications were available, CV events were also evaluated at the longest follow-up time point (Table 3). The proportion of CV events in the DRV/r-treated patients was 0.18% (9/4992). For DRV/r, the incidence rate (IR) was 1.44 per 1000 person-years.

**Table 3:** Cardiovascular events reported in clinical trials containing darunavir.

Author	Study	Weeks considered in the meta-analysis	CV AE/SAE	Other weeks in the same study	CV AE/SAE
Aberg^46^	METABOLIK	48	no CV AE/SAE		
Mills^5^	ARTEMIS	96	no CV AE/SAE	192	1 stroke in the DRV arm; 1 MI in the LPV/r arm
Hamzah^53^	MIDAS	48	no CV AE/SAE		
Gianotti^49^	2PM	48	no CV AE/SAE		
Maggiolo^56^	PROBE	48	no CV AE/SAE		
Raffi^47^	NEAT 001	96	no CV AE/SAE		
Clotet^22^	POWER 1–2	48	no CV AE/SAE	96* (1-2-3)	no CV AE/SAE
Molina^45^	FLAMINGO	96	no CV AE/SAE	48	1 MI in the DRV/r arm; 1 cardiomyopathy in the DTG arm
Martinez^44^	ATADAR	48	no CV AE/SAE		
Madruga^9^	TITAN	48	no CV AE/SAE		
Slama^19^	IMEA	48	no CV AE/SAE		1 pericarditis in the ATV arm
Chéret^48^	OPTIPRIM	96	no CV AE/SAE		
Lennox^37^	ATG5257	96	no CV AE/SAE		
Huhn^50^	DRIVESHAFT	48	no CV AE/SAE		
Clumeck^55^	MONET	96	no CV AE/SAE	144	no CV AE/SAE
Valantin^21^	MONOI	96	4 CV SAE	48	3 CV AE grades 3-4
Guaraldi^54^	MONARCH	48	no CV AE/SAE		
Santos^52^	LOPIDAR	48	no CV AE/SAE		
Nishijima^58^	SPARE	48	no CV AE/SAE		
Cahn^8^	ODIN	48	no CV AE/SAE		
Girard^57^	PROTEA	96	no CV AE/SAE		

*Publication at 96 weeks including POWER Studies-1-2-3

Abbreviations: CV = cardiovascular; AE = adverse event; ATV = atazanavir; DTG = dolutegravir; DRV = darunavir; LPV = lopinavir; MI = Myocardial Infarction; SAE = serious adverse event

### Mono vs triple therapy

**Figure 6:**
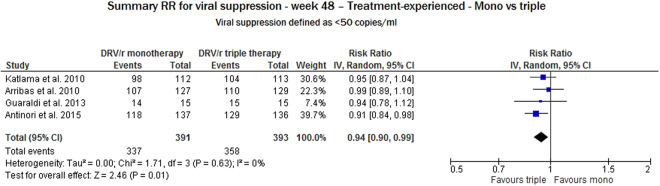
Meta-analysis of viral suppression for ART-experienced subjects at 48 weeks of follow-up considering monotherapy vs triple therapy.

To evaluate the sole impact of DRV/r on safety, we compared the results of monotherapy with those of triple therapy in the studies reporting on DRV/r in treatment-experienced, virologically controlled subjects. The monotherapy arm of the trials was taken as a comparator. We considered four studies reporting endpoints of viral suppression at week 48. DRV/r was significantly better in triple therapy than in monotherapy (RR 0.94, 95% CI: 0.90–0.99). No heterogeneity was found between the estimates (I^2^ = 0%, p = 0.63) (Fig. 6).

**Figure 7:**
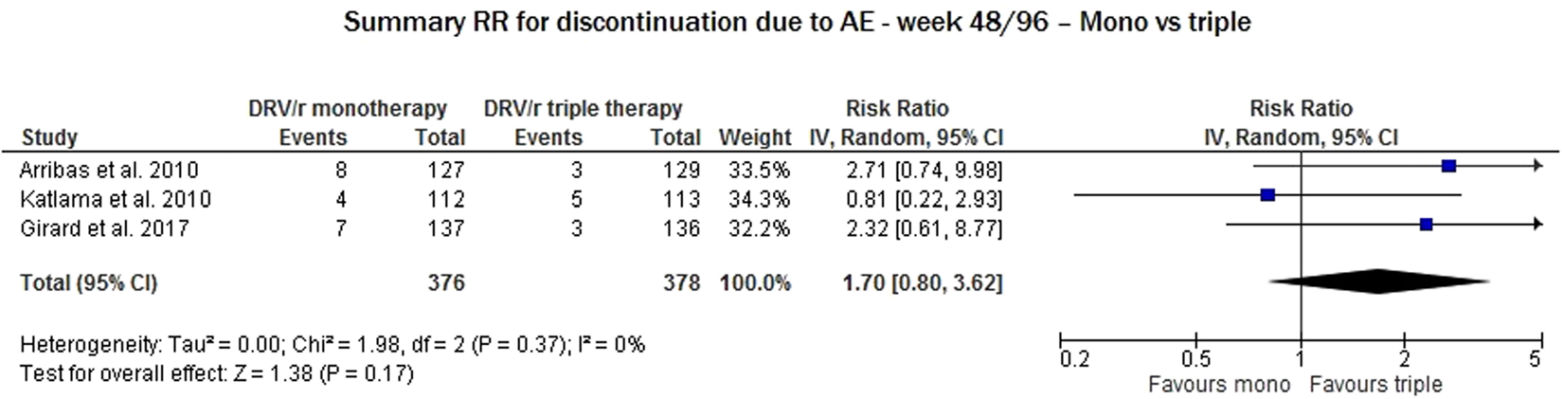
Meta-analysis of studies reporting data on any serious adverse events (AEs) considering monotherapy vs triple therapy* - *Favors triple indicates that a higher number of AEs was reported in the triple therapy arms.

We evaluated three studies to assess discontinuation due to AEs at week 48. This variable did not significantly differ between DRV/r in monotherapy and DRV/r in triple therapy (RR 1.70, 95% CI: 0.80–3.62) in the absence of heterogeneity between RRs (I^2^ = 0%, p = 0.37) (Fig. 7).

## Discussion

Nineteen RCTs were included in this meta-analysis. The first RCT was published in 2007 and described treatment-experienced subjects, and the most recent trials were published in 2016 and involved naive subjects.

In the ITT analysis of the ART-naïve subjects, the virological response rate did not differ between the DRV/r and the comparator arms, at both 48 and 96 weeks, despite the wide variability of the studies. Heterogeneity can be explained by the baseline characteristics of the subjects included in studies, such as ARTEMIS^10^ and IMEA^19^ compared to FLAMINGO^20^. The subjects were more advanced in ARTEMIS^10^ and IMEA^19^ than in FLAMINGO^20^, with higher viral loads and lower CD4+ cell counts.

In the ITT analysis at week 48 of the ART-experienced failing subjects, the virological response rate was significantly higher for DRV/r than for the comparator drugs, regardless of the previous clinical and treatment history and despite the wide heterogeneity of the studies. To date, DRV/r is the only antiretroviral drug which have been studied in highly pretreated subjects, and this population has not been enrolled in any subsequent study. In a pooled analysis of POWER studies^22^, DRV/r provided a sustained virological response in patients with reverse transcriptase and protease resistance-associated mutations at baseline^22^. This finding shows the high potency and high genetic barrier of DRV/r^23^ and its efficacy against resistant viruses. These results are in line with the well-known genetic barrier of DRV/r and its proven efficacy against resistant viruses. Furthermore, the DRV genetic barrier is still unequalled with respect to both other PIs and to inhibitors of strand transfer (INSTIs).

In the ITT analysis at week 48 of the ART-experienced virologically controlled subjects, the virological response rate was comparable to that of the comparator group (I^2^ = 34.6%, p = 0.122). In four of these studies, DRV/r was used as a monotherapy, and its potency in reaching viral undetectability was confirmed, as was its good penetration in HIV reservoirs^21,23–25^. These results were achieved in clinical practice in both naïve and highly experienced patients, the latter of whom had approximately seven years of FU, as reported in an Italian observational cohort (the TMC114HIV4042 study^13^, registered in ClinicalTrials.gov under the identifier NCT01375881).

The safety profile of DRV/r was similar to that of the comparator irrespective of the dosage and the comparator used. In this analysis, pooling the naïve and experienced subjects could have introduced bias, because the naïve subjects had never taken DRV/r. Notably, in the FLAMINGO trial, significantly more SAEs occurred in the DTG arm than in the DRV/r arm (RR 0.58; 95% CI: 0.35–0.97)^20^.

The safety data were also confirmed in the TMC114IHIV4042 study^13^, where the DRV/r-based treatment was well tolerated, with only 3.0% of the treatment discontinuations due to AEs. Notably, no differences were observed in the AE/SAE types and/or frequencies in this study compared to those reported in the DRV/r RCTs^1,2,13^.

Moreover, following the recently presented D:A:D cohort data on cardiovascular risk in HIV-positive subjects treated with DRV/r-based regimen^26^, we showed that the cardiovascular events rates in all studies included in this meta-analysis were low, even though the observational period was approximately three years compared to the more than six-year observation period included in the D:A:D^26^.

Triple therapy proved to be superior in efficacy (defined as viral suppression) to monotherapy. Patient characteristics (i.e., residual viremia and a nadir CD4+ count <100 cells/μL) should be taken into account when establishing a monotherapy regimen, as highlighted by Gianotti *et al*.^27^, who reported selection criteria for entry of candidate virologically suppressed HIV-positive individuals into DRV/r monotherapy^27^. Following this scoring system, DRV/r monotherapy and standard therapy “could be equally effective” with the same virological failure rate as standard triple therapy^27^.

No mutations associated with DRV resistance were reported for monotherapy based on DRV/r, and sensitivity to DRV was maintained^28–30^. To date, no INSTI drug has shown the same genetic barrier: INSTI resistance-associated mutations have been found in failing monotherapy^31^. In terms of safety, adverse events leading to therapy discontinuation were relatively rare and were even rarer in the monotherapy studies^28–30^.

### Limitations

The limitations of this meta-analysis include the use of different comparators in the studies, inhomogeneity in the study duration, the use of different timepoints for the efficacy/safety assessments, the wide timespan of the studies considered and the inclusion of only English-language publications. All the RCTs included were open-label; therefore, the risk of performance bias was increased. However, the outcomes evaluated were objective measures, which might have decreased the risk of bias. Furthermore, this analysis only included studies using DRV boosted with the pharmaco-enhancer ritonavir. However, the results of two recent registrative studies conducted with naïve and virologically suppressed, experienced patients taking ART based on DRV boosted with the new pharmaco-enhancer cobicistat have been published^32,33^. Further research including those data are recommended.

### Strenghts

The main strength of this meta-analysis is the comprehensive search for published clinical studies from multiple electronic databases using a cross-checking strategy for additional potentially missed articles. The meta-analytic approach allowed us to obtain more precise estimates of the pooled results, which can provide clinicians with suggestions for use in clinical practice, as previous meta-analyses have done^34,35^. Furthermore, the studies considered here were conducted in different years; therefore, the patient characteristics differed greatly among the studies (in previous years, the patients were more advanced). Nevertheless, the results shown in response to DRV treatment were consistent and confirmed its well-known efficacy and safety profile; thus, this treatment remains an effective option for current patients.

Using this meta-approach, we re-analyzed study-level data; however, additional original studies involving a longer follow-up period and patients enrolled in real-life settings are required to better understand the efficacy, effectiveness and safety of DRV/r.

## Conclusion

The evidence shown in this analysis confirms that DRV/r is an effective regimen for ART-naive and ART-experienced subjects, with no differences from the comparator arms detected. DRV/r was safe and well-tolerated in every group of subjects. The good safety profile of DRV when used in monotherapy is highlighted.

## Methods

### Search strategy

A systematic literature search of clinical trials including DRV use in HIV-positive patients was conducted in September 2016 using the Medline and EMBASE databases. No data were generated in this work, which analyzed publicly available publications. We did not prepare a specific review protocol for this project. We adopted a wide-ranging search strategy using a predefined generic search string with no temporal restrictions and no search filters whenever possible. This strategy was finalized to minimize the probability of excluding relevant papers from the present meta-analysis. The Medline/Pubmed search string was as follows: “(darunavir OR prezista OR tmc114) AND trial”. A similar combination of keywords was used in the EMBASE search; however, that search was restricted to clinical trials using the “study types” filter. A cross-check for additional articles that were potentially missed during the main search process was conducted by exploring the Cochrane Register of Controlled Trials (CENTRAL) and Google Scholar (using the same keywords and reviewing the first 150 papers according to their relevance) and performing thorough searches of the reference lists of relevant reviews and the papers selected for inclusion. Figure 1 provides a flow-chart with detailed information on the search and selection processes.

### Inclusion and exclusion criteria

The identified publications were considered for inclusion in the meta-analysis if the following criteria were met: randomized clinical trials with at least 48 weeks of follow-up and with DRV use in at least one study arm. Observational studies, interventions other than DRV use, reviews, meta-analyses, indirect comparisons, commentaries and other articles lacking original data were excluded. Single-arm trials and pooled analyses were also excluded after careful consideration. Conference abstracts were included, whereas unpublished studies and articles in languages other than English were excluded. No studies were excluded *a priori* for weakness of design or data quality.

### Study selection, data extraction and risk of bias assessment

Two researchers independently examined the articles retrieved from the Medline/PubMed and EMBASE databases. Discrepancies between the researchers’ results were discussed and resolved. In the first selection step, the articles were evaluated based on their titles and abstracts. After merging the publications from the PubMed and EMBASE searches, a total of 134 unique publications remained. The second and third selection steps were based on full-text examinations of the retrieved articles. Sixty articles reporting data on the efficacy or safety of DRV in HIV-positive patients from RCTs with at least 48 weeks of follow-up were retained. Fourteen of these studies were included in the tables but were not used in the meta-analyses due to the relatively small number of studies with their specific characteristics (i.e., they reported results for follow-up periods other than 48 or 96 weeks or they reported results from trials on treatment-experienced subjects with switched or mixed treatments).

Two researchers reviewed the selected studies and extracted relevant information. In particular, the extracted data included the trial name, enrollment period, geographic area, number of patients included and treatment regimen in each study arm, the reason for discontinuation of earlier treatments (for studies with treatment-experienced patients), the patient characteristics at baseline, and the follow-up duration. This information was organized in two tables that separated the trials with treatment-naïve and treatment-experienced patients. The latter patients were further divided into subgroups representing trials of (i) treatment-experienced failing subjects treated with a DRV 600 mg BID regimen compared with another regimen, (ii) treatment-experienced virologically controlled subjects treated with a DRV 800 mg regimen compared with another regimen, and (iii) treatment-experienced subjects treated with a mixed/other DRV regimen. The main results for the efficacy (i.e., viral suppression defined as <50 copies/ml) and safety outcomes (i.e., treatment discontinuation due to adverse events or serious adverse events) were also extracted into spreadsheets for subsequent meta-analyses. Whenever available, we extracted the results from the intention-to-treat analysis. Discrepancies between researchers were checked in the original reports and resolved.

The risk of bias in the included studies was assessed by three authors using the Cochrane risk of bias tool^36^. Discrepancies between the researchers were discussed and resolved through discussion with a senior reviewer.

### Statistical analyses

In the efficacy outcome analyses, the results obtained at weeks 48 and 96 and for the treatment-naïve and treatment-experienced patients were always analyzed separately. However, for the safety outcomes, all trials were jointly analyzed using the results for the longest follow-up time when several results were available from the same trial. The risk ratios for each study were pooled. When the risk ratio was not provided but sufficient data were available in the publication to compute this measure, we calculated unadjusted risk ratios and their 95% CIs from the outcome distributions of subjects in the treatment and control arms. When more than one publication reported results from the same study (i.e., with extended follow-up periods), we included the earliest publication in the meta-analysis because the completion rate was higher and the endpoint was more similar to those of the other studies. The ACTG5257 study^37^ was a three-arm trial. Therefore, we pooled data from the ATV and RAL arms to compute a single risk ratio for each efficacy and safety outcome. These ratios were included in the meta-analysis.

We computed summary risk ratios (RR) for each efficacy and safety outcome for the patients treated with DRV compared to other treatments using random-effects models (i.e., as weighted averages using the inverse of the sum of the variance of the log (risk ratio) and using the moment estimator of the variance between studies as the weight)^38,39^. Heterogeneity between trials was assessed using the χ^2^ test (defined as a p-value less than 0.10), and inconsistency was measured using the I^2^ statistic, which describes the percentage of total variation across studies due to heterogeneity rather than chance^40^. Values of the I^2^ statistic of approximately 25%, 50% and 75% are indicative of low, moderate and high heterogeneity, respectively^40^. The presence of publication bias was assessed based on a visual examination of the funnel plots and by applying the tests proposed by Begg and Mazumdar^41^ and Egger^42^. We conducted sensitivity analyses by excluding each study one by one from the meta-analysis. No other sub-group analyses were planned. All statistical analyses were performed using the RevMan software (version 5.3 for Windows).

## Electronic supplementary material

Supplementary Figure
