# Supplementary material for: Efficacy and safety of boosted darunavir-based antiretroviral therapy in HIV-1-positive patients: results from a meta-analysis of clinical trials
Source: Sci Rep. 2018 Mar 27;8:5288. doi: 10.1038/s41598-018-23375-6 (PMC5869729; doi:10.1038/s41598-018-23375-6)
Supplement: Supplementary file 1 — Supplementary Figure [file 41598_2018_23375_MOESM1_ESM.pdf]

Efficacy and safety of boosted darunavir-based antiretroviral therapy in HIV-1-positive patients: results from a meta-analysis of clinical trials

Antinori A[1], Lazzarin A[2], Uglietti A[3], Palma M[3], Mancusi D[3*] and Termini R[3]

1. HIV/AIDS Department, National Institute for Infectious Diseases "Lazzaro Spallanzani" IRCCS, Roma, Italy
2. Infectious Diseases, San Raffaele Scientific Institute, Milano, Italy
3. Janssen-Cilag SpA, Medical Affairs Department, Infectious Diseases, Cologno Monzese (MI), Italy

*dmancusi@its.jnj.com

Supplementary Figure 1 – Risk of bias for the included studies

	Random sequence generation (selection bias)	Allocation concealment (selection bias)	Blinding of participants and personnel (performance bias)	Blinding of outcome assessment (detection bias)	Incomplete outcome data (attrition bias)	Selective reporting (reporting bias)	Other bias
Aberg JA et al, 2012	+	+	−	−		+	−
Antinori A et al, 2015	+	+	−	−	+	+	+
Arribas JR et al, 2010	+	+	−	−	+	+	+
Banhegyi D et al, 2012	+	+	−	−	+	+	+
Cahn P et al, 2011	+	+	−	−		+	+
Chéret A et al, 2015	+	+	−	−	+	+	
Clotet B et al, 2014	+	+	−	−	+	+	−
Clotet et al, 2007	+	+	−	−	+	+	+
Gianotti N et al, 2016	+	+	−	−	+	+	
Girard PM et al, 2017	+	+	−	−	+	+	+
Guaraldi G et al, 2014	+	+	−	−	+	+	
Hamzah L et al, 2015	+	+	−	−		+	
Huhn GD et al, 2015	+	+	−	−	+	+	−
Katlama C et al, 2010	+	+	−	−	+	+	+
Lennox JL et al, 2014	+	+	−	−	+	+	+
Madruga JV et al, 2007	+	+	−	−	+	+	+
Maggiolo F et al, 2016	+	+	−	−	+	+	+
Martinez E et al, 2014	+	+	−	−		+	
Mills AM et al, 2009	+	+	−	−	+	+	+
Molina JM et al, 2015	+	+	−	−	+	+	−
Moltó J et al, 2015	+	+	−	−		+	
Nishijima et al 2013	+	+	−	−		+	
Ortiz R et al, 2008	+	+	−	−	+	+	+
Santos JR et al, 2016	+	+	−	−	+	+	+
Slama L et al, 2016	+	+	−	−		+	

Legend:

Green = Low risk

Red = High risk

Empty cell = Unclear risk
